# Catalytic Ozonation of Nitrobenzene by Manganese-Based Y Zeolites

**DOI:** 10.3389/fchem.2020.00080

**Published:** 2020-02-12

**Authors:** Jingze Hu, Yiming Li, Shaoshuai Nan, Brandon A. Yoza, Yifan Li, Yali Zhan, Qinghong Wang, Qing X. Li, Shaohui Guo, Chunmao Chen

**Affiliations:** ^1^State Key Laboratory of Petroleum Pollution Control, China University of Petroleum-Beijing, Beijing, China; ^2^Hawaii Natural Energy Institute, University of Hawaii at Manoa, Honolulu, HI, United States; ^3^Department of Molecular Biosciences and Bioengineering, University of Hawaii at Manoa, Honolulu, HI, United States

**Keywords:** ozonation, catalytic ozonation, Y zeolites, Mn oxides, wastewater treatment

## Abstract

Catalytic ozonation process (COP) is considered as a cost-efficient technology for the treatment of refractory chemical wastewaters. The catalyst performance plays an important role for the treatment efficiency. The present study investigated efficiencies and mechanisms of manganese (Mn)-based Y zeolites in COPs for removing nitrobenzene from water. The catalysts of Mn/NaY and Mn/USY were prepared by incipient wetness impregnation, while Mn-USY was obtained by hydrothermal synthesis. Mn-USY contained a greater ratio of Mn^2+^ than Mn/NaY, and Mn/USY. Mn oxides loaded on Y zeolites promoted the COP efficiencies. Mn/NaY increased total organic carbon removal in COP by 7.3% compared to NaY, while Mn/USY and Mn-USY increased 11.5 and 15.8%, respectively, relative to USY in COP. Multivalent Mn oxides (Mn^2+^, Mn^3+^, and Mn^4+^) were highly dispersed on the surface of NaY or USY, and function as catalytic active sites, increasing mineralization. Mn-USY showed the highest total organic carbon removal (44.3%) in COP among the three catalysts, because Mn-USY had a higher ratio of Mn^2+^ to the total Mn oxides on the surface than Mn/NaY and Mn/USY and the catalytic effects from intercorrelations between Mn oxides and mesoporous surface structures. The hydroxyl radicals and superoxide radicals governed oxidations in COP using Mn-USY. Nitrobenzene was oxidized to polyhydroxy phenol, polyhydroxy nitrophenol, and p-benzoquinone. The intermediates were then oxidized to small organic acids and ultimately carbon dioxide and water. This study demonstrates the potential of Y zeolites used in COP for the treatment of refractory chemical wastewaters.

## Introduction

Nitrobenzene is an electrophilic and refractory environmental contaminant that contains a nitro group and a benzene ring. Nitrobenzene is listed as a potential carcinogen by the National Institute of Environmental Health Sciences (Wang and Ma, 2018). Nitrobenzene is removed from water by adsorption (Dasgupta et al., 2018), reduction (Li Y. et al., 2018), and oxidation (El Metwally et al., 2019). The catalytic ozonation process (COP) is widely studied for its application toward the removal of refractory organic chemicals (ROCs) from water. This process is easy to manage, highly efficient, and safe (Chen et al., 2018). The use of catalysts facilitates the decomposition of ozone into highly active species of oxygen, including hydroxyl radicals (·OHs), superoxide radicals (${\text{O}}_{2}^{\cdot -}$) and singlet oxygen radicals (^1^O_2_) (Zhao et al., 2009; Wang et al., 2019). Many catalysts have been previously studied to treat refractory environmental contaminants. Those include natural minerals (Chen et al., 2015), Al_2_O_3_ and ZSM-5 loaded with metal oxides (Chen et al., 2017, 2018; Xu et al., 2019), and waste sludge biochar (Chen et al., 2019). The use of zeolites catalysts (Nawrocki and Kasprzyk-Hordern, 2010), including those that contain active metallic components (Rosal et al., 2010), have been investigated. However, the catalytic mechanisms that are involved with the degradation of organic chemicals in water remain unclear. ZSM-5 zeolites adsorb ozone and organic molecules onto its surface, promoting proximal reactions (Ikhlaq et al., 2013). For example, Fe-SBA-15 was found to adsorb oxalic acid, which is then oxidized by radical species (Yan et al., 2016). Acid-treatment of natural zeolite has been determined to promote the removal of methylene blue mainly via ·OHs mediated oxidation (Valdes et al., 2012).

Dealuminated Y zeolite (USY) was initially recognized as an effective catalyst for use with the COP treatment of phenol (Dong et al., 2008). USY facilitates ozone decomposition and ·OHs generation (Dong et al., 2008). The Y zeolites possessing a large specific surface area can effectively disperse metal oxides (Vu et al., 2018). While the utilization of Y zeolites for the treatment of volatile organic compounds in gases has been well-documented, little attention has been given to the COP treatment of ROCs in water (Kwong et al., 2008; Einaga et al., 2011). The USY zeolite is produced by the application of an ammonium ion exchange and dealumination by NaY (Sato et al., 1999; Santikunaporn et al., 2004). NaY and USY are differentiated by its textures, structures, and the molar ratio of Si to Al. These differences influence catalytic performance (Sato et al., 2001). To further improve catalytic performance, the surface loading of metallic oxides (Jeirani and Soltan, 2017) such as manganese (Mn) is widely used (Sui et al., 2011; Sun et al., 2014).

In the present study, loadings of Mn oxides on NaY and USY were studied for the catalytic ozonation efficiencies. In addition, Mn loaded Y zeolites (Mn/NaY, Mn/USY, and Mn-USY) were compared with NaY and USY to understand the catalytic mechanisms of the Mn loaded Y zeolites for the reduction of nitrobenzene in water.

## Experimental

### Materials

The NaY zeolite was purchased from Nanjing Xianfeng Nanomaterials Technology Co., Ltd., China. Sodium bicarbonate (NaHCO_3_; 99.5 wt.%), ammonium chloride (NH_4_Cl; 99.5 wt.%), manganese nitrate solution (Mn(NO_3_)_2_; 50 wt.%) and potassium hydroxide (KOH; 99.5 wt.%) were all obtained from Beijing Chemical Reagents Co., Ltd., Beijing, China. Methanol (CH_3_OH; 99.9 wt.%), p-benzoquinone (C_6_H_4_O_2_; 99.5 wt.%) and methylene chloride (CH_2_Cl_2_; 99.9 wt.%) were purchased from Fisher Scientific. Inc. 5,5-Dimethyl-1-pyrroline (DMPO) was purchased from Sigma-Aldrich. Inc. Ultrapure water (18.2 mΩ/cm) was produced by a Direct-Pure UP ultrapure water system (Rephile Shanghai Bioscience Co., Ltd., Shanghai, China).

### Preparation of Catalysts

One hundred grams of NaY was washed by 500 mL of ultrapure water three times and then dried prior to use. Forty grams of NaY was exchanged using 1 mol/L NH_4_Cl solution in a mass ratio of 1:10 (solid-liquid ratio) at an initial pH value at 3–3.5. After stirring at 90°C for 90 min and filtering, the obtained solid sample was dried at 90°C for 4 h and calcined at 550°C for 4 h. After a repeated operation, the USY catalyst was obtained. Mn/NaY and Mn/USY were prepared according to the incipient wetness impregnation method. Briefly, an aliquot of 1.3 mL of 50 wt.% Mn(NO_3_)_2_ solution was added to 8.0 g water followed by impregnation of 10 g NaY or USY, then dried at 90°C for 4 h and calcined at 550°C for 4 h. Mn-USY was obtained by hydrothermal synthesis. Mn(NO_3_)_2_ and CO(NH_2_)_2_ were blended in a molar ratio of 1:4 in 20 mL deionized water. This mixture was further blended with 10 g of USY and stirred at 80°C for 90 min and then the sample was placed in a crystallization kettle and heated to 100°C for 4 h under slow rotation. Afterwards, the sample was filtered, washed, dried, and calcined at 550°C for 4 h. Mn oxides (MnO_x_) were synthesized according to the same procedure, but without USY. The contents of Mn on Mn-based Y zeolites were detected by ICP-AES. 0.1 g of catalyst was dissolved in a mixture of 5 mL HNO_3_ and 1 mL HF, and saturated H_3_BO_3_ solution was added to eliminate the interference of F^−^. The three zeolites (Mn/NaY, Mn/USY, and Mn-USY) contained a similar content (3.0 wt.%) of Mn.

### Characterization of Catalysts

X-ray powder diffraction (XRD) was recorded on XRD-6000 powder diffraction instrument (Shimadzu, Kyoto, Japan) with a 40.0 kV working voltage and 40.0-mA electric current. Crystallinities of the Y samples were calculated from the eight defined peak areas (2 θ = 15.6, 18.6, 20.3, 23.6, 27.0, 30.7, 31.3, and 34°) with the NaY standard sample as a reference according to the ASTM standards (D3906-03). The pore structures were determined with an ASAP 2000 accelerated surface area and porosimetry system (Micromeritics, Norcross, GA, USA). An Inductively Coupled Plasma-Atomic Emission Spectrometer (ICP-AES) (Perkin Elmer DV7000, Waltham, MA, USA) was used for determining the actual Mn content in Mn-based Y zeolites. X-ray photoelectron spectroscopy (XPS) results were obtained on a PHI Quantera SXM photoelectron spectrometer (ULVAC-PHI, Chanhassen, MN, USA) using Al Ka radiation (h*v* = 1486.6 eV). The binding energy (BE) values were referred to the C1s line at 284.8 eV. Atomic ratios in the XPS sampling region were evaluated by peak area integration. The surface morphology and element compositions were examined on a Quanta 200 F scanning electron microscope (SEM) and a Tecnai G2 F20 transmission electron microscope (TEM) (FEI, Hillsboro, OR, USA) with an energy-dispersive X-ray (EDX) spectroscope (FEI, Hillsboro, OR, USA). H_2_-temperature-programmed reduction (H_2_-TPR) was performed on an AutoChem II 2920 (Micromeritics, USA) with 10 vol.% H_2_ in Ar. A Magna-IR 560 ESP FT-IR spectrometer (Nicolet, Madison, WI, USA) was used for identifying the functional groups on the surface. The diffuse reflectance spectra were recorded on a U-4100 UV-Vis spectrophotometer (Hitachi, Japan) with an integrated sphere diffuse reflectance attachment. The powder samples were loaded in a transparent quartz cell and UV-Vis absorbance values were measured in the region of 200–800 nm at room temperature. The reflectance of the standard support was used as the baseline for the relative catalyst measurement.

### Ozonation of Refractory Organic Chemicals

Nitrobenzene is one of typical ROCs in chemically contaminated wastewaters. In the present study, nitrobenzene at a concentration of 100 mg·L^−1^ in ultrapure water was used to determine catalytic activity. The initial total organic carbon (TOC) and pH value of nitrobenzene solution were 55 mg·L^−1^ and 5.89, respectively.

The COP experiments were performed with a system (Figure S1) that consists of a 40 L of oxygen tank (Beijing Jinggao Gas Co., Ltd., Beijing, China), a COM-AD-02 ozone generator (Anseros Asvanced Oxidation Technologies Co., Ltd., Tübingen-Hirschau, Germany), two GM-6000-OEM ozone gas analyzers (Anseros Asvanced Oxidation Technologies Co., Ltd., Tübingen-Hirschau, Germany), a D07-7 mass flow controller coupled with a D08-1F flow readout box (Beijing Sevenstar Flow Co., Ltd., Beijing, China), a 800 mL quartz column reactor and an exhaust gas collector. The reactor was placed on a ZNCL-BS intelligent magnetic stirrer (Shanghai Kankun Instrument Equipment Co., Ltd., Shanghai, China) at 700 rpm·min^−1^ to promote mass transfer among nitrobenzene, ozone and catalysts. During COP experiments, an aliquot of 500 mL of nitrobenzene solution and 0.5 g of catalyst was added in the reactor at 25°C. The gaseous ozone was then introduced through a porous diffuser at the bottom of the reactor having a flow rate of 7.5 mg·min^−1^. After treatment, nitrogen gas was sparged into the nitrobenzene solution at a rate of 3.0 L·min^−1^ to quench the reaction. Fifteen milliliter of treated solution was extracted into a 40 mL of syringe when sampling. The solution in the syringe was then filtered through a 0.45 μm end filter (Tianjin Jinteng Experimental Equipment Co., Ltd., Tianjin, China) to remove catalyst particles before further analysis. The filtered catalyst was dried and recycled for repetitive experiments. The lost amount of the catalyst was supplemented to 0.5 g by addition of the fresh catalyst (~ 5% in average) prior to the next use. After reaction, the concentration of Mn ion in solution was detected by ICP-AES at 259.37 nm for the quantification of Mn. Single adsorption process and single ozonation process (SOP) experiments were performed with the same experimental system as COP. Controls did not have ozone or a catalyst. All adsorption, SOP, and COP experiments were performed in triplicate.

The radicals quenching experiments were performed for identification of the oxidation mechanism. The ·OHs scavenger NaHCO_3_ (0.5 and 1.0 g·L^−1^), methanol (5 mg·L^−1^) and the ${\text{O}}_{2}^{\cdot -}$ scavenger p-benzoquinone (5 mg·L^−1^) were added into nitrobenzene solution prior to experiments.

Electron spin resonance (ESR) experiments were performed on a Bruker ElexSys E500 spectrometer to directly identify radical species. The test was performed under −183°C (90 K), the operating conditions were centerfield: 3,520 G; sweep width: 200 G; microwave frequency: 9.057 GHz; modulation frequency: 100 GHz; power: 10.00 mW. For radical analysis, 5,5-dimethyl-1-pyrroline (DMPO) was employed as the spin-trapping agents. DMPO was used for capture ·OHs alone, and DMPO with methyl alcohol was used for capture ${\text{O}}_{2}^{\cdot -}$.

Nitrobenzene and degradation intermediates were identified by a Gas Chromatograph-Mass Spectrometer (GC-MS, 7890 B/MS, Agilent Technologies), a High Performance Liquid Chromatograph (HPLC, Ultimate 3,000, Thermo Scientific) and an Ion Chromatograph (IC, Dionex IC2100, Thermo Scientific). When GC-MS was used for analysis, CH_2_Cl_2_ was the extraction solvent. A DB-35 capillary column (30 m × 0.25 mm × 0.25 μm) was used. The initial temperature was 30°C, held for 2 min, increased to 45°C at a rate of 10°C/min and kept for 5 min. It was then heated up to 150°C at a rate of 2°C/min, and kept at 150°C for 5 min, then heated up to 225°C at 2°C/min, and kept at 225°C for 5 min. HPLC was equipped with a Hypersil ODS-C18 column (150 × 4.6 mm × 5 μm), and using a mixture of methanol and ultra-pure water (volume ratio was 4:6) as the mobile phase. The flow rate was 0.8 mL/min. The temperature was maintained at 35°C. The concentration of nitrobenzene and intermediates were detected at 254 and 280 nm, respectively (Zhang and Ma, 2008). Ion chromatographic separation of small organic acids was accomplished on an AG11-HC column (4 × 250 mm). The mobile phase was KOH solution (30 mmol/L). The injection volume was 25 μL. The flow rate was 1.0 mL/min.

## Results and Discussion

### Characteristics of Catalysts

**Figure 1:**
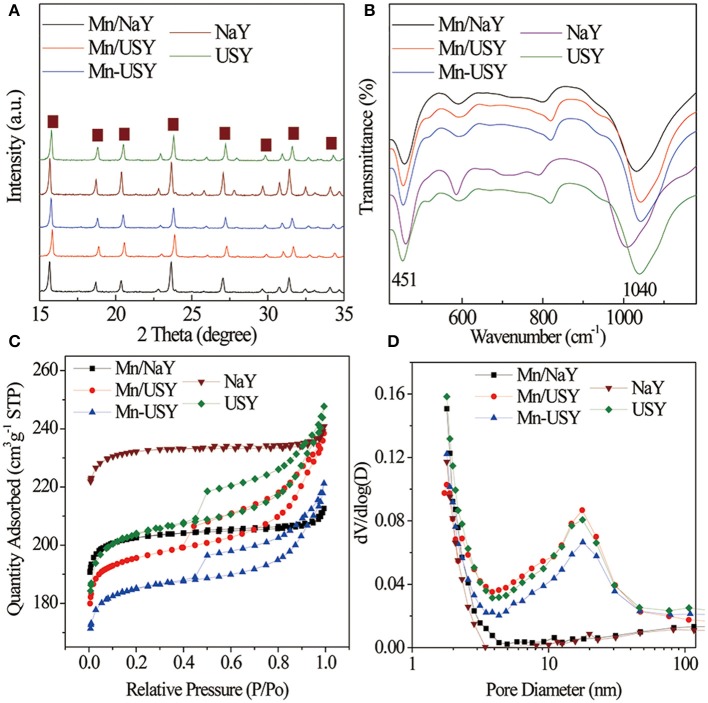
XRD spectra **(A)**, FT-IR **(B)** spectra, adsorption-desorption isotherms **(C)**, and pore distributions **(D)** of Mn-based Y zeolites.

The XRD peak patterns for both the Y zeolites and Mn-based Y zeolites (Figure 1A) were similar, and have the typical characteristic peaks associated with the FAU type Y zeolite (Li R. et al., 2018). The relative crystallinities were as follows: Mn/NaY (90%), Mn/USY (73%) and Mn-USY (77%), NaY (100%), and USY (81%). Significant crystallinity differences were observed between NaY and USY. Dealumination of the NaY (Si/Al molar ratio at 3.19) results in a partial destruction of the resultant USY (Si/Al, 5.16) skeleton structures, reducing its crystallinity. Introduction of Mn oxides into Y zeolites also results in a slight decrease of relative crystallinity. XRD diffraction peaks from Mn oxides were not observed for the Mn-based Y zeolites, due to surface dispersion, low concentration and/or small size. Negligible but observed changes in the FT-IR spectra of the Y zeolites were noted after loading Mn oxides. A typical peak at about 1,040 cm^−1^ (Figure 1B) is attributed to the asymmetrical stretching of Si–O–Si and Al–O–Si bending vibrations in the Y zeolites (Lutz et al., 2010; Asadi et al., 2018). The peak at 451 cm^−1^ is related to the Si–O and/or Al–O vibrational bending of the internal tetrahedral aluminosilicate framework (Zhao et al., 2016; Li et al., 2019). The typical absorption peak at 510 cm^−1^ due to Mn–O stretching (Naidja et al., 2002) was not observed, due to its masking from the stronger signals produced by the Al–O–Si.

According to IUPAC classification, the adsorption-desorption isotherms of NaY and Mn/NaY exhibited a typical micropore structure (Figure 1C). The isotherms for USY, Mn/USY, and Mn-USY zeolites suggest mixed micropore-mesopore structures due to the partial dealumination of Y zeolites (Denayer and Baron, 1997). The USY, Mn/USY, and Mn-USY had an additional peak at 20 nm in pore distribution in comparison with NaY and Mn/NaY (Figure 1D). High mesopore areas and pore volumes were observed in USY zeolites relative to NaY zeolites (Table 1). The surface area and pore volume of Y zeolites decrease after loading the Mn oxides because the formed Mn oxides occupy the surface and block the micropores of the Y zeolites (Yang et al., 2019). The differences in surface area and pore volume for the Mn/USY (624 m^2^/g and 0.37 cm^3^/g) and Mn-USY (588 m^2^/g and 0.34 cm^3^/g) are related to the Mn oxides morphology as surface dispersion.

**Table 1:** Zeolites pore structures determined by N_2_ adsorption-desorption and surface element contents by XPS.

**Zeolites**	**NaY**	**USY**	**Mn/NaY**	**Mn/USY**	**Mn-USY**
**Pore structures by N_2_ adsorption-desorption (surface area: m^2^·g^−1^; pore volume: cm^3^·g^−1^)**					
Total surface areas	739	652	646	624	588
Micropore surface areas	706	585	604	565	538
Mesopore surface areas	33	67	42	59	50
Total pore volumes	0.37	0.38	0.33	0.37	0.34
Micropore volumes	0.34	0.28	0.30	0.27	0.26
Mesopore volumes	0.03	0.10	0.03	0.10	0.08
**Surface element contents by XPS**					
Mn2p weight %			5.03	2.02	1.81
Mn^2+^/Mn^3+^/Mn^4+^			0.20/0.39/0.41	0.33/0.32/0.35	0.43/0.36/0.21

**Figure 2:**
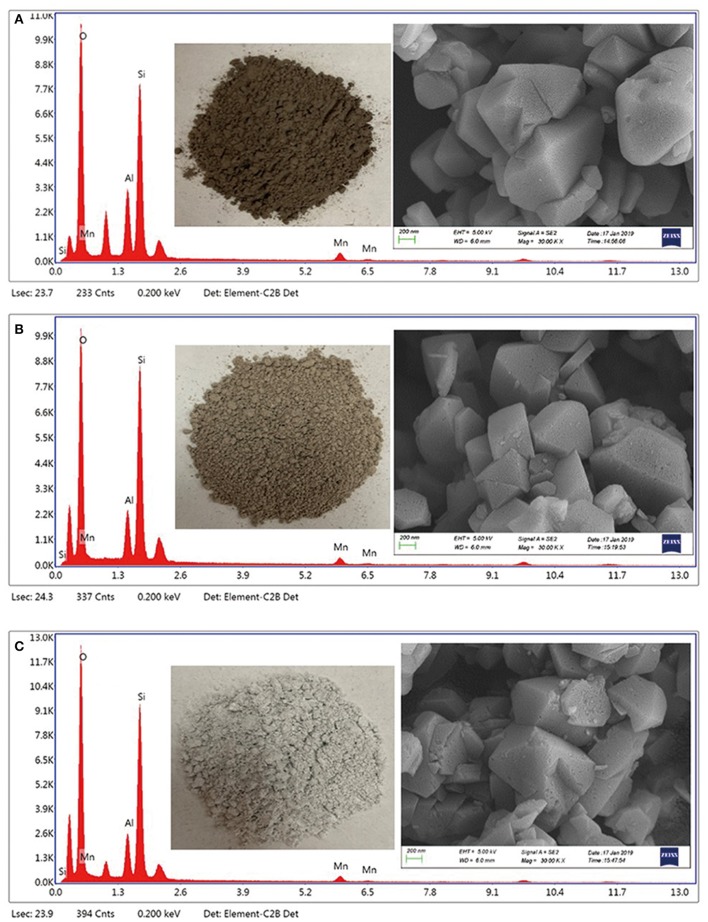
SEM images-EDX spectra of Mn/NaY **(A)**, Mn/USY **(B)**, and Mn-USY **(C)**.

Cubic particles about 400–600 nm were observed on the Y zeolites surface (Figure 2 and Figure S2), which are typical for FAU types of zeolite (Zhang et al., 2019). A microanalysis performed using EDS confirmed the presence of the Mn oxides. Interestingly, the Mn/NaY (Figure 2A), Mn/USY (Figure 2B), and Mn-USY (Figure 2C) varied visually and were dark brown, light brown and blue gray, respectively. The NaY (Figure S2A) and USY (Figure S2B) were white.

**Figure 3:**
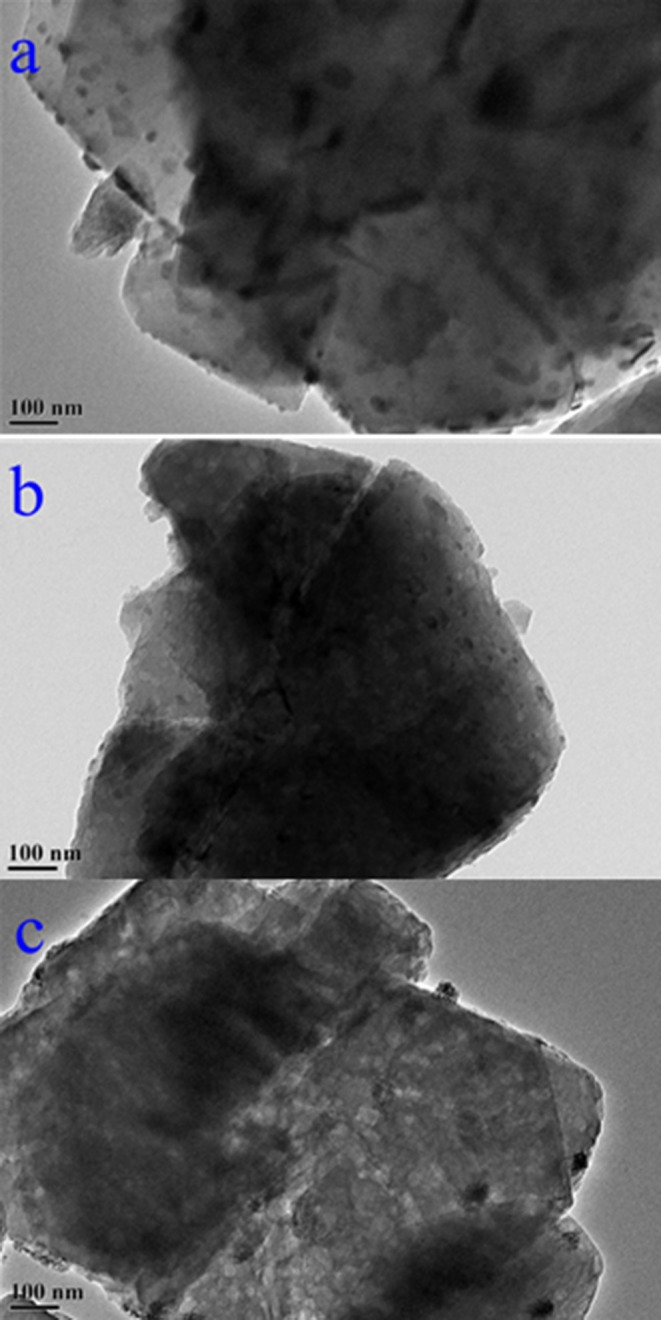
TEM images of Mn/NaY**(a)**, Mn/USY**(b)** and Mn-USY**(c)**.

The weighted Mn percentages, defined by EDX were: Mn/NaY (5.05%), Mn/USY (3.11%), and Mn-USY (2.75%). The darker colored Mn/NaY is related to the high surface content of the distributed Mn, and the valency of the oxides. The Mn oxides that were deposited on the surfaces of the Y zeolites formed irregularly shaped micro-agglomerates. The surface Mn oxides were clearly observed according to TEM images of Mn/NaY (Figure 3a) and Mn/USY (Figure 3b) compared to NaY (Figure S3A) and USY (Figure S3B). The Mn oxides on the Mn-USY are unclear in the TEM image (Figure 3c). The USY catalysts exhibit clear mesoporous surface structures by TEM.

**Figure 4:**
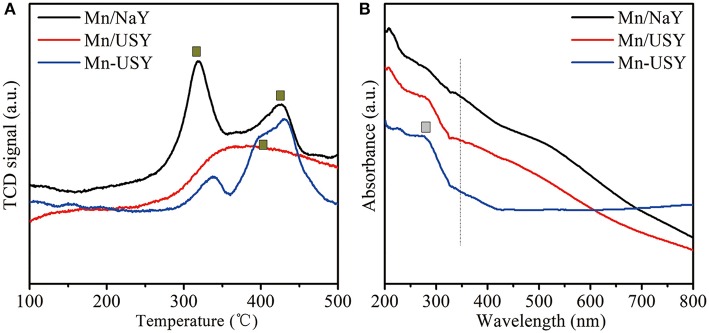
H_2_-TPR **(A)** and UV-vis **(B)** spectra of Mn/NaY, Mn/USY, and Mn-USY.

The peaks reduced between 200 and 500°C were observed by H_2_-TPR profiles for Mn/NaY, Mn/USY, and Mn-USY (Figure 4A). The Mn/NaY display two well-developed peaks at 200–350 and 350–500°C, while the Mn/USY has a broad undefined peak in 200–500°C. The Mn-USY has a weak peak at 200–350°C and a broad peak at 350–500°C. During the process of hydrogen reduction, the Mn oxides are reduced in two steps: MnO_2_ or Mn_2_O_3_ → Mn_3_O_4_ and Mn_3_O_4_ → MnO (Li et al., 2013; Wu et al., 2015). The different multivalent states of Mn oxides coexist with the catalysts. Additionally, the relative peak area at 200–350°C was higher than that at 350–500°C for Mn/NaY, while the opposite trend was observed for Mn-USY. The peak areas for Mn/USY fell between the other two catalysts. Mn having higher valency exists in the Mn/NaY. The reduced peak temperature for the Mn/NaY suggests a weaker interaction between the support and Mn oxides.

UV-Vis spectra for Mn/NaY, Mn/USY, and Mn-USY were generated between the range of 200–800 nm (Figure 4B). The Mn/USY had an absorption at 200–330 (L1) nm and another broad adsorption (L2) between 350 and 650 nm. The absorption profile is associated with a charge transfer (CT) that from O^2−^ → Mn^2+^ and O^2−^ → Mn^4+^ and Mn^3+^, respectively (Lamaita et al., 2005; Stamati et al., 2007). The Mn/NaY displayed a weaker L1 and an enhanced L2, while Mn-USY exhibited an enhanced L1 and a weakened L2. This observation suggests a greater Mn^2+^ ratio in Mn-USY than that in Mn/NaY and Mn/USY.

**Figure 5:**
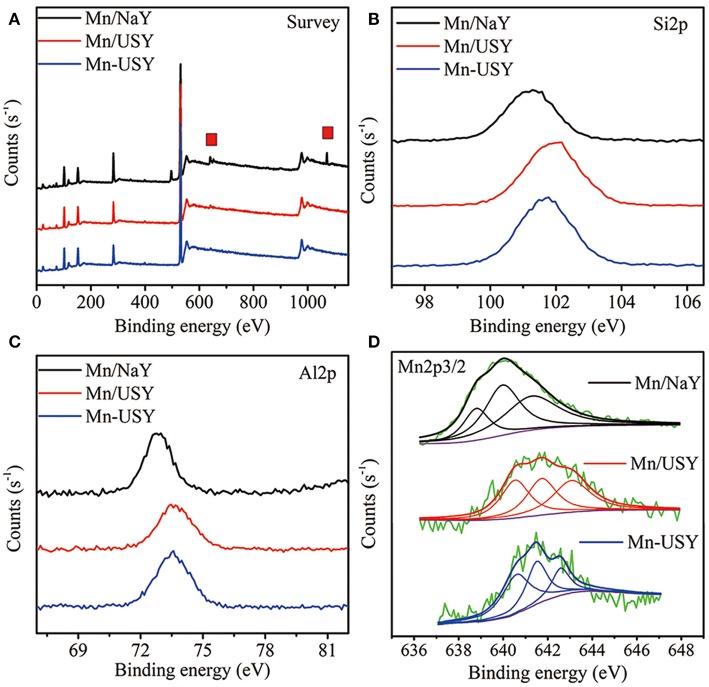
Survey **(A)**, Si2p **(B)**, Al2p **(C)** and Mn2p **(D)** XPS spectra of Mn/NaY, Mn/USY and Mn-USY.

XPS was used to further determine Mn distribution and valency. The XPS spectra for Mn/USY and Mn-USY indicate lower content of Na (binding energy at about 1,070 eV) relative to Mn/NaY. This is likely resulted from the removal of Na^+^ during ${\text{NH}}_{4}^{+}$ exchange (initial pH value at 3–3.5) for USY preparation (Figure 5A). The weight of Na for the Mn/NaY, Mn/USY, and Mn-USY was 7.36, 0.32, and 0.27%, respectively. A strong elemental Mn peak (binding energy at about 640 eV) was observed in Mn/NaY relative to the Mn/USY and Mn-USY, suggesting high surface distribution of Mn oxides. The Mn2p weight was 5.03% for Mn/NaY, 2.02% for Mn/USY, and 1.81% for Mn-USY. The binding energies of Si2p (Figure 5B) and Al2p (Figure 5C) were at ~101–102 and 72–74 eV, attributable to Si^4+^ (Plymale et al., 2015) and Al^3+^ (Hadnadjev et al., 2008), respectively. Broad peaks are associated with Al2p XPS for Mn/USY and Mn-USY. The four-coordinate Al framework is partially transformed to five-coordinate Al and non-framework Al during preparation of USY from NaY (Wang et al., 2014). Mn/NaY have low intensity XPS Si2p peaks compared to Mn/USY and Mn-USY, and identical XPS Al2p observed among the catalysts. The surface molar ratio of Si to Al (Si/Al) was 2.7, 4.3, and 3.6 for Mn/NaY, Mn/USY, and Mn-USY, respectively. The observable peak shifts of XPS Si2p and Al2p are related to slight differences in the chemical coordination environment. The XPS spectra from Mn2p are centered between 640–643 and 650–653 eV, and are from the Mn2p_3/2_ and Mn2p_1/2_ (Zhang et al., 2015; Figure 5D). According to Mn2p_3/2_ peaks fitting results, multivalent Mn oxides (Mn^2+^, Mn^3+^, and Mn^4+^) are formed in all the catalysts (Dai et al., 2012). The multivalent Mn in Mn/NaY has the highest Mn^4+^ (0.41) content when compared to Mn/USY (0.35) and Mn-USY (0.21). The multivalent Mn in Mn-USY has the highest Mn^2+^ (0.43) contents relative to Mn/NaY (0.20) and Mn/USY (0.33). Black or brown coloration is observed for the MnO, MnO_2_, MnOOH, Mn_2_O_3_, and MnO(OH)_2_, a light color is seen with Mn(OH)_2_ (white). The valence state (Figure 5) and visual color difference (Figure 2) suggest that more Mn(OH)_2_ (white) is distributed in the Mn-USY catalyst (Figure 2C). The different properties for the catalysts observed are resulted from the differences in support material and the methods used to load Mn oxides.

### Catalytic Efficiencies

**Figure 6:**
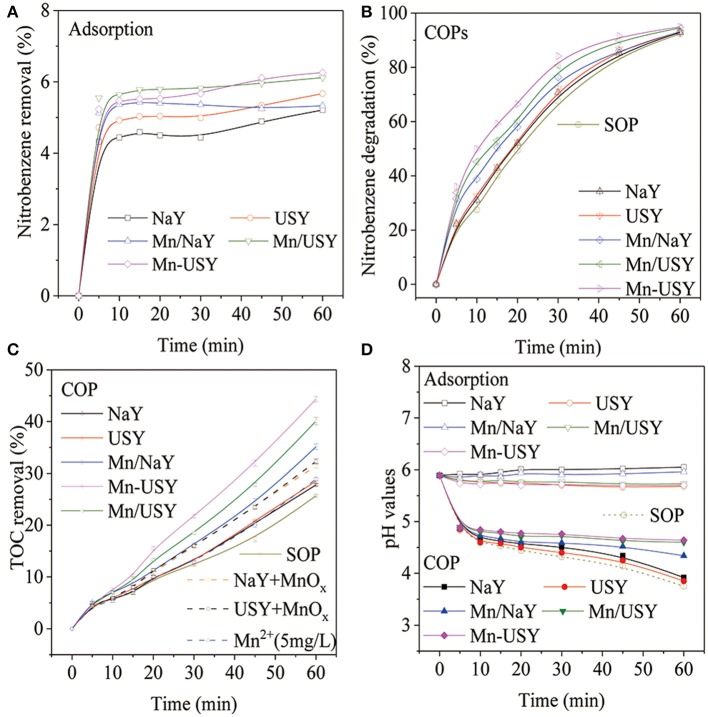
Nitrobenzene removal by adsorption **(A)**, nitrobenzene degradation after COPs **(B)**, TOC removal after COPs **(C)**, and pH changes **(D)** with catalysts (0.5 g catalyst, ozone 7.5 mg·min^−1^, initial solution pH 5.89, reaction temperature 25°C and 60 min treatment).

The adsorption of nitrobenzene in solution quickly reached saturation (4–6%) for all Y zeolites (Figure 6A). The Y zeolites exhibited weak adsorption toward nitrobenzene, and slight adsorption capacity differences were observed for the NaY, USY and their Mn-based catalysts. In the previous report using ZSM-5 as catalysts during COP, NaZSM-5 (high Na^+^) had weaker adsorption toward nitrobenzene in solution when compared with HZSM-5 (low Na^+^) (Chen et al., 2018). These results that were influenced by the Na^+^ content were not observed with the Y zeolites. The COPs using NaY, USY, Mn/NaY, Mn/USY, and Mn-USY, degraded 31.0, 32.7, 38.8, 44.2, and 49.9% of the nitrobenzene, respectively, 10 min after treatment. In contrary, the SOP degraded 29.5% of the nitrobenzene (Figure 6B). Due to catalytic activity of Mn-based Y zeolites, the nitrobenzene degraded after 10 min by COPs was higher than the total sums of SOP and adsorption. Increased activity related with catalysis was however not observed with NaY and USY. The catalytic efficacy between the COPs and SOP gradually decreased with time. After a 60 min treatment, the COPs and SOP degraded identical amounts of nitrobenzene (92–96%).

As determined by TOC, 25.7% of the nitrobenzene was removed after a 60 min treatment using SOP (Figure 6C). Including NaY and USY in the COPs reduced TOC by 27.8 and 28.5% of TOC after 60 min treatment. The NaY and USY promote a small increase in TOC that is attributable to the weak adsorption. Using the Mn/NaY, Mn/USY, and Mn-USY as catalysts during COP increases the TOC removal. After a 60 min of treatment, the TOC was reduced by 35.1, 40.0, and 44.3%, respectively. The greater difference in TOC removals cannot be attributed to adsorption (2–4%) (Figure S4) and is therefore a realized catalytic influence. The TOC removed by COPs using Mn-based Y zeolites is consistently greater than SOP. The oxidized nitrobenzene produces oxalic acid intermediates (Zhao et al., 2008b) that are further mineralized during COPs with Mn-based Y zeolites, but not with SOP or when NaY and USY are used.

Within 10 min, the pH values of nitrobenzene solution rapidly decreased after the initiation of SOP or COPs, followed by a slow decrease over the treatment time (Figure 6D). Higher pH values were observed with the COPs using Mn-based Y zeolites when compared with COPs containing NaY or USY, and also compared with SOP. Adsorption has little to no influence on pH values. The COPs and SOP generate acidic intermediates that decrease solution pH values (Zhao et al., 2008a). The Mn-based Y zeolites used during COP further mineralize acidic intermediates, which reduces acidity.

Catalytic activity with the NaY and USY was not observed. The catalytic activities of 4A and ZSM-5 during COP were previously determined (Chen et al., 2018; Ikhlaq et al., 2018). The types of zeolites and the specific ROCs have an influence on catalytic performances during COPs. The Mn-based Y zeolites are catalytically active, which may be attributed to the multivalent Mn oxides (Mn^2+^, Mn^3+^, and Mn^4+^) (Sun et al., 2014; Huang et al., 2017) or the Mn irons in solution (Gracia et al., 1998; Andreozzi et al., 2001). The leaching of Mn irons from catalysts during ozonation was determined by ICP-AES and the concentrations of Mn irons were 4.25, 3.38, and 1.75 mg·L^−1^ in 60 min of COP with Mn/NaY, Mn/USY and Mn-USY, respectively, after first run. In order to verify the role of Mn ions and Mn oxides in the COP of nitrobenzene, 0.015 g of MnO_x_ and 11 μL of Mn(NO_3_)_2_ solution were added into the ozonation system, respectively. The TOC removal in ozonation of nitrobenzene with Mn^2+^ was 28.88% (Figure 6C), slightly higher than single ozonation (25.67%). When NaY and USY were mixed with solid MnOx, both “MnO_x_ + NaY” and “MnO_x_ + USY” showed approximately 32% of TOC removal in ozonation of nitrobenzene (Figure 6C), which was slightly lower than the COP with Mn/NaY (33.2%). The experimental results suggest that MnO_x_ is the main factor promoting catalytic ozonation of nitrobenzene with Mn-based Y zeolites. The Mn/NaY has relatively low catalytic activity when compared with Mn/USY and Mn-USY. The Mn/NaY did not have high activity during COP, although it had a high concentration of surface distributed Mn. Overly concentrated Mn oxides on the surface can result in aggregation decreasing available active sites (Qi et al., 2012; Chen et al., 2017). It has been previously demonstrated that mesoporous supports can improve the interactions between metallic oxides and facilitate the adsorption of ozone during COPs (Zhuang et al., 2014; Ryu et al., 2019). The mesoporous USY facilitates proximal reactions with the Mn oxides and ozone relative to the microporous NaY. Different methods used for the loading the metal oxides can result in different catalytic performances (Bing et al., 2012). This may plausibly explain why the Mn/USY catalyst exhibits lower catalytic activity in comparison with the Mn-USY catalyst. Additionally, a lower oxide state of Mn facilitates ozone decomposition (Ryu et al., 2019). The highest activity observed for Mn-USY may be also attributed to its high ratio of Mn^2+^.

The leaching of Mn ions (5.83%) from Mn-USY after 60 min reaction was detected by ICP-AES. The reusability of Mn-USY after repeated uses during COP treatment was examined. TOC removal of COP with Mn-USY decreased gradually with the increase of repeated times due to the leaching of Mn from the catalyst. When the number of runs increased from 3 to 4, TOC removals of COP with Mn-USY decreased to <35%, and gradually approaching to the TOC removal in SOP after 5 runs. The results showed Mn-based Y zeolites had a weak-moderate stability in COPs of pollutants, which warrants further investigations to improve the stability of Mn oxides on Y zeolites.

### Mechanisms

**Figure 7:**
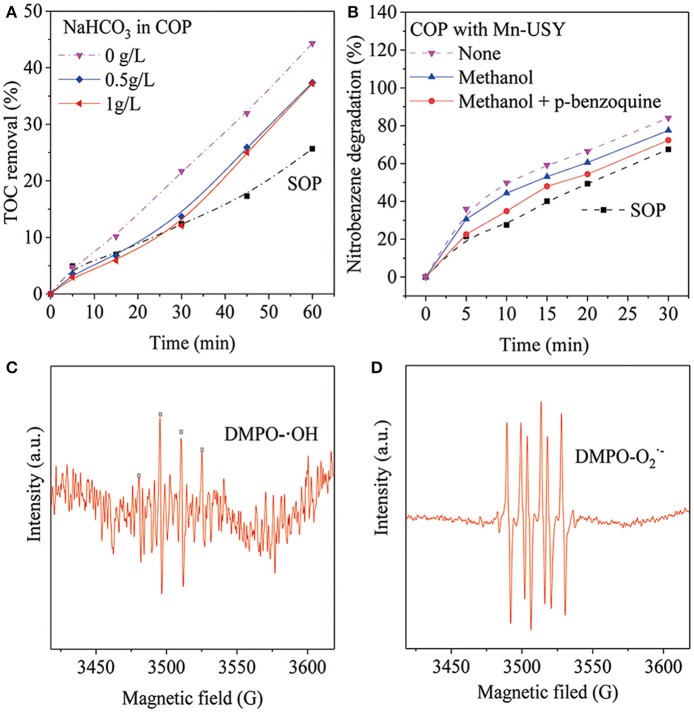
TOC reduction after COP treatment (60 min) using Mn-USY with the addition of NaHCO_3_ **(A)**, methanol (5 mg·L^−1^) and p-benzoquinone (5 mg·L^−1^) **(B)**, liquid-phase ESR spectra of ·OHs **(C)** and ${\text{O}}_{2}^{\cdot -}$ **(D)** with DMPO spin trapping.

The introduction of NaHCO_3_ was performed to conifirm whether the generation of ·OHs promoted the removal of TOC during COP with Mn-USY (Ma and Graham, 2000). Methanol and p-benzoquinone were used to study the roles of the oxygen species in COP with Mn-USY (Khataee et al., 2017; Asgari and Salari, 2019). NaHCO_3_ (0.5 g·L^−1^) significantly reduced the TOC removal during COP with Mn-USY (Figure 7A), which suggests that ·OH mediates oxidation to remove TOC. When a higher concentration of NaHCO_3_ (1.0 g·L^−1^) was used, changes in TOC removal were not observed, suggesting that the ·OH generated was mostly consumed. The TOC removal with 1.0 g·L^−1^ of NaHCO_3_ in the COP using the Mn-USY was higher than by SOP. The other active oxygen species (other than ·OHs) coexist during COP. This contributes toward the mineralization of nitrobenzene in solution. The degradation of nitrobenzene significantly decreased to 77.5% upon addition of methanol (5 mg·L^−1^) (Figure 7B). After addition of both methanol (5 mg·L^−1^) and p-benzoquinone (5 mg·L^−1^), the degradation of nitrobenzene dropped to 72.4% at 30 min. The experimental results showed that ·OHs and ${\text{O}}_{2}^{\cdot -}$ promote the degradation of nitrobenzene. ESR was performed to directly detect the radicals and it does prove this. The DMPO-·OH peak (Figure 7C) has an intensity ratio of 1:2:2:1 (Fang et al., 2009) and DMPO- ${\text{O}}_{2}^{\cdot -}$ (Figure 7D) is peaked at 1:1:1:1 (Zhao et al., 2018), further confirming the production of ·OH and ${\text{O}}_{2}^{\cdot -}$ during COP with Mn-USY. The multivalent Mn oxides (Mn^2+^, Mn^3+^, and Mn^4+^) in Mn-USY promotes the generation of ·OHs and ${\text{O}}_{2}^{\cdot -}$ by the transferring electrons between the metallic oxides and ozone molecules.

### Proposed Pathways for Nitrobenzene Degradation

**Figure 8:**
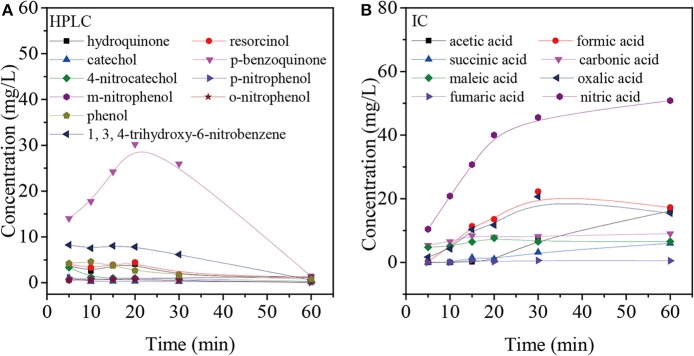
Intermediate products based on HPLC **(A)** and IC **(B)** from the degradation of nitrobenzene using COP with Mn-USY.

In order to better understand the pathways of nitrobenzene degradation during COP, the intermediates formed during the degradation of nitrobenzene by COP using Mn-USY were determined by GC-MS, HPLC, and IC. Figure 8 shows that p-nitrophenol, m-nitrophenol, o-nitrophenol, phenol, resorcinol, hydroquinone, catechol, p-benzoquinone, 4-nitrocatechol, 1, 3, 4-trihydroxy-6-nitrobenzene, nitrate, carbonate, oxalic acid, formic acid, acetic acid, succinic acid, maleic acid, and fumaric acid were generated during COP. The production of phenol indicated the occurrence of denitration. The nitro group can be removed from nitrobenzene by a nucleophilic substitution reaction with ozone and ${\text{O}}_{2}^{\cdot -}$, and the unstable intermediates were then further oxidized to phenol (Wang et al., 2015; Yang et al., 2018). Alternatively, the ·OHs can transfer electron forming phenyl radicals that can be in turn transformed into phenol (Zhao et al., 2008a). The phenolic hydroxyl group belongs to the electron-donating group, ozone molecules and ·OHs tend to react with the ortho- and para-positions of hydroxyl to form catechol and hydroquinone. With further oxidation, p-benzoquinone and 1, 2, 4-trihydroxybenzene are formed prior to ring opening. The accumulation of p-benzoquinone concentration increases to its maximum after 20 min, confirming this.

Moreover, ·OHs could easily react with the α-, β-, or/and γ-carbons of nitrobenzene by electrophilic addition to form o-nitrophenol, m-nitrophenol, and p-nitrophenol, respectively (Bhatkhande et al., 2003). During the COP, the concentration of p-nitrophenol, m-nitrophenol and o-nitrophenol is low, indicating that the electrophilic radicals may continue to attack p-nitrophenol, m-nitrophenol and o-nitrophenol. Similarly, the reaction can occur preferentially in the ortho- and para- positions of phenolic hydroxyl groups (Goi et al., 2004). The ·OHs attack the carbon atoms attached to the nitro group and nitrate ions are released by the radical addition. The ·OHs electrophilic addition occurs continuously to form resorcinol, hydroquinone, catechol, 4-nitrocatechol and 1,3,4-trihydroxy-6-nitrobenzene.

**Figure 9:**
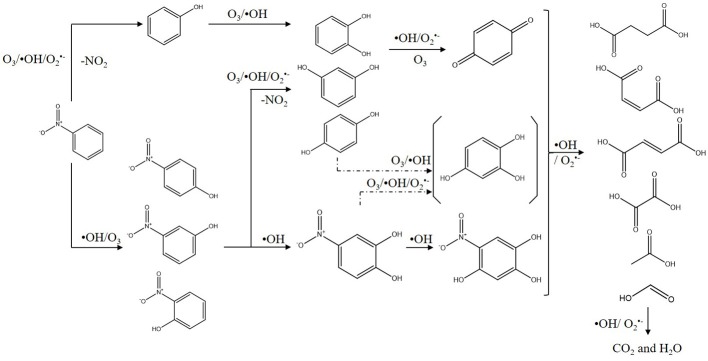
Proposed degradation pathways of nitrobenzene using COP with Mn-USY (All compounds were detected, except the one in the parenthesis).

The above-mentioned intermediates will further react with ·OHs forming carboxylic acid such as formic acid, acetic acid and oxalic acid. These small molecules are finally mineralized to CO_2_ and H_2_O. In addition, the accumulation of nitrate indicates that the COP has been accompanied by the reaction of denitration (Figure 9).

## Conclusions

The catalysts of Mn-based Y zeolites were investigated for catalytic ozonation efficiencies and mechanisms during the COP treatment of nitrobenzene in water. The multivalent Mn oxides (Mn^2+^, Mn^3+^, and Mn^4+^) are highly dispersed on the surface of NaY or USY, and function as catalytically active sites that increase mineralization. The Mn-USY removed the most TOC during COP which is related to its high surface ratio of Mn^2+^, and coordinated interaction between the Mn oxides and mesoporous structures. The oxidation is mediated by ·OHs and ${\text{O}}_{2}^{\cdot -}$, contributing to the TOC removal. These catalytic mechanistic results are broadly applicable to catalyst design for use with the COP treatment of ROCs in water.

## Data Availability Statement

The datasets generated for this study are available on request to the corresponding author.

## Author Contributions

JH and CC conceived and designed the experiments. JH, SN, and YimL performed the experiments. JH, YZ, QW, BY, and SG interpreted and analyzed the data. YifL and SN contributed reagents, materials, analysis tools. BY, QL, CC, and JH. wrote the manuscript.

### Conflict of Interest

The authors declare that the research was conducted in the absence of any commercial or financial relationships that could be construed as a potential conflict of interest.

## Supplementary Material

Figure S1 Schematic representation of the experimental setup.

Figure S2 SEM images and EDX spectra of NaY **(a)** and USY **(b)**.

Figure S3 TEM images of NaY **(a)** and USY **(b)**.

Figure S4 TOC removal in adsorption process with Y zeolites.
