# Supplementary figures and images for: Catalytic Ozonation of Nitrobenzene by Manganese-Based Y Zeolites
Source: Front Chem. 2020 Feb 12;8:80. doi: 10.3389/fchem.2020.00080 (PMC7028746; doi:10.3389/fchem.2020.00080)

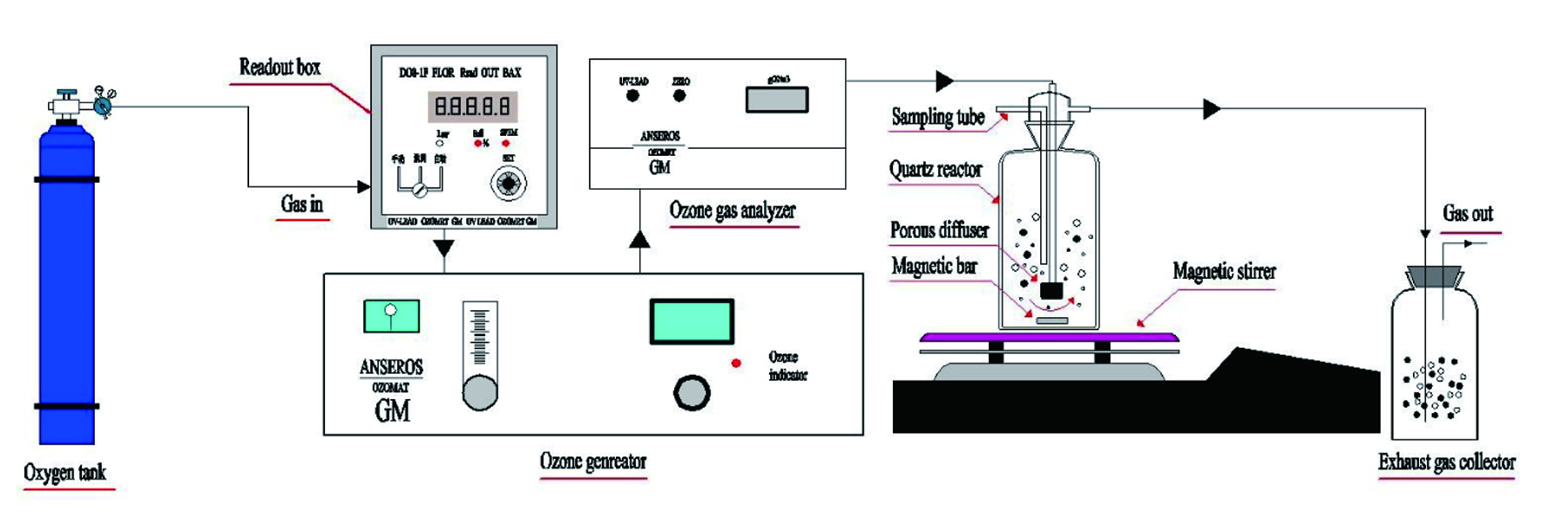

Supplement: Figure S1 — Schematic representation of the experimental setup. [file Image_1.TIF]

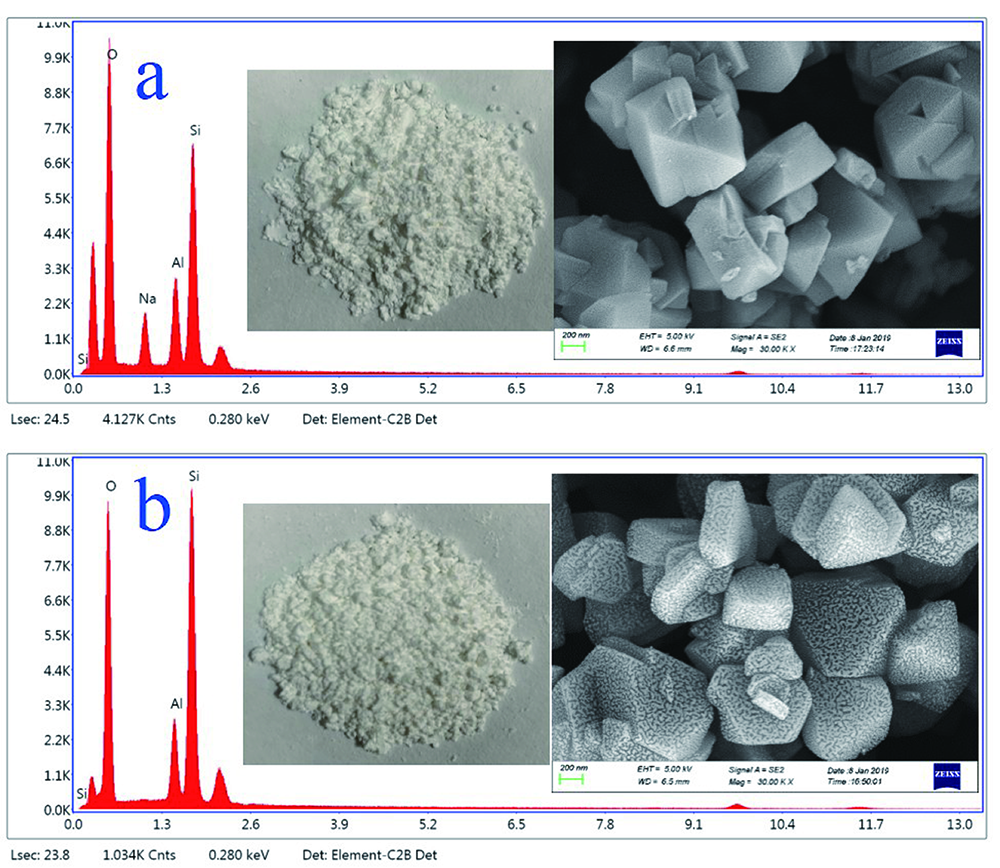

Supplement: Figure S2 — SEM images and EDX spectra of NaY (a) and USY (b). [file Image_2.TIF]

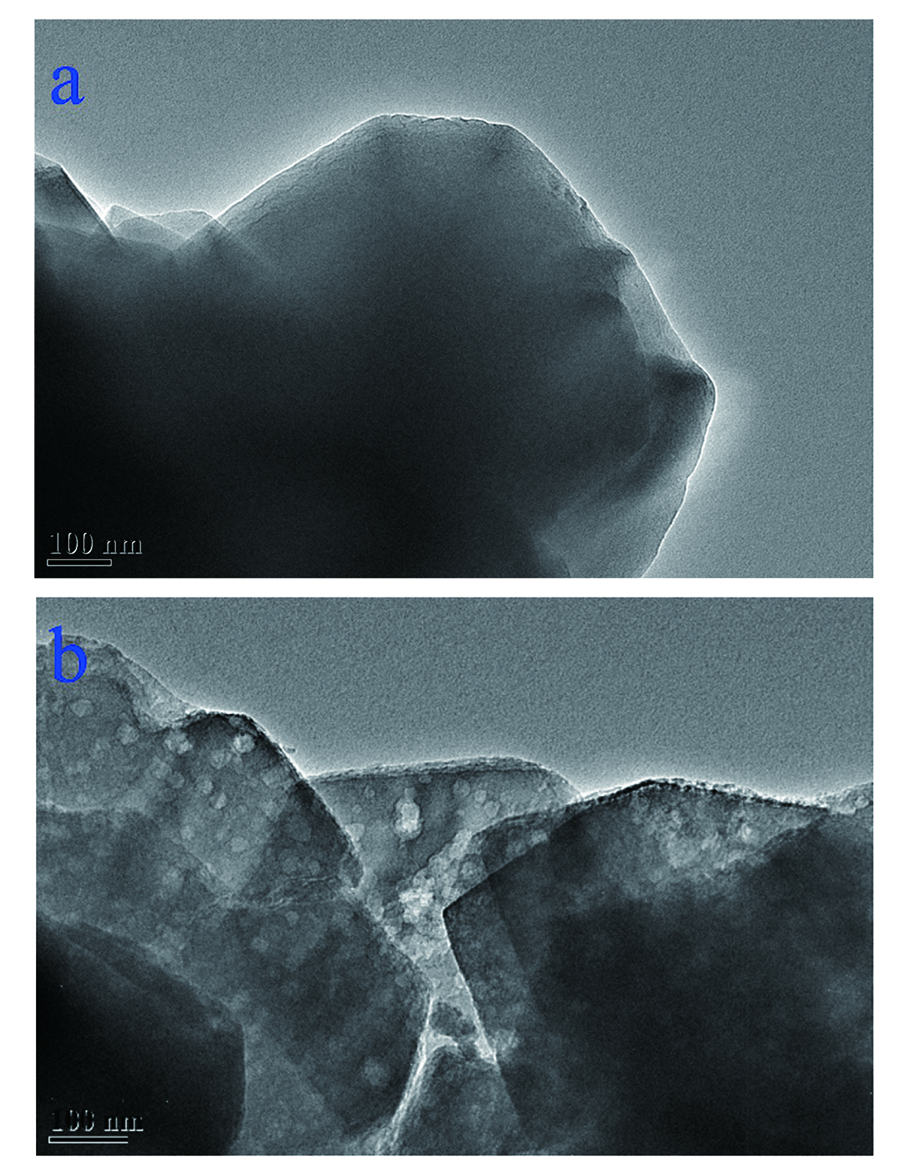

Supplement: Figure S3 — TEM images of NaY (a) and USY (b). [file Image_3.TIF]

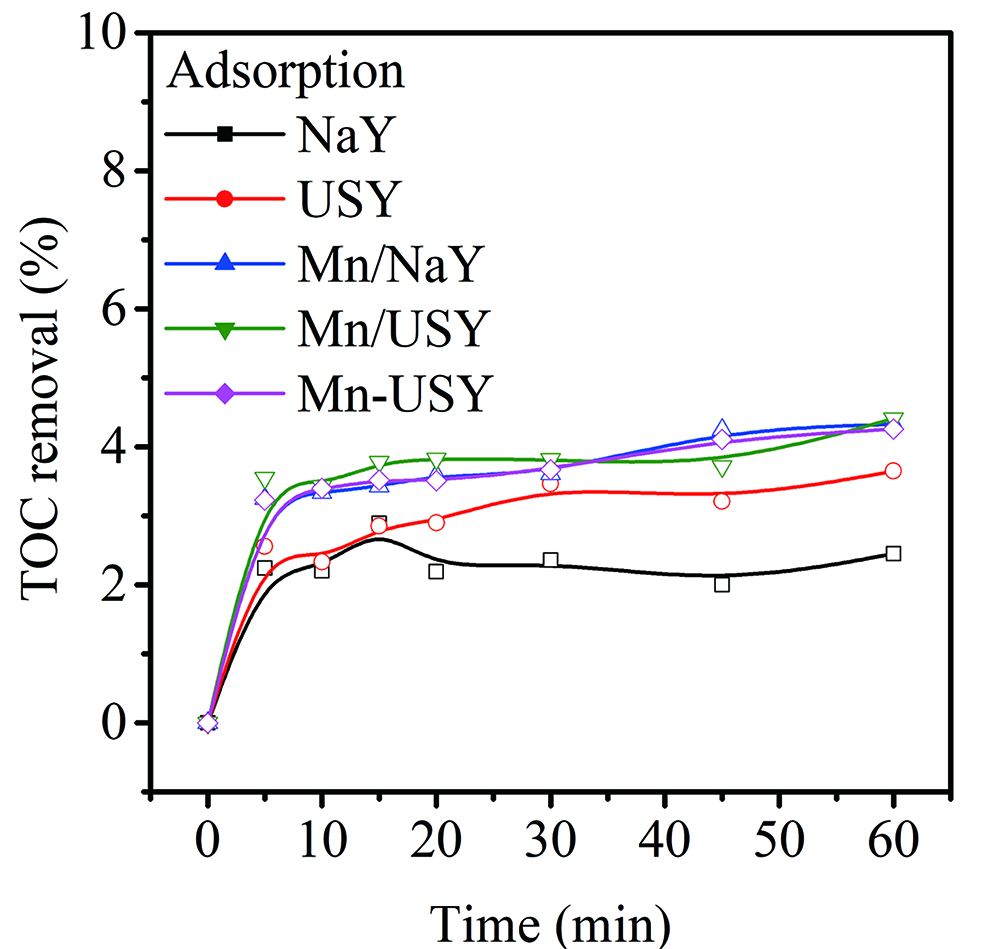

Supplement: Figure S4 — TOC removal in adsorption process with Y zeolites. [file Image_4.TIF]
